# Stakeholder Perceptions About Group B *Streptococcus* Disease and Potential for Maternal Vaccination in Low- and Middle-Income Countries

**DOI:** 10.1093/cid/ciab794

**Published:** 2021-11-03

**Authors:** Carsten Mantel, Thomas Cherian, Melissa Ko, Stefano Malvolti, Elizabeth Mason, Michelle Giles, Philipp Lambach

**Affiliations:** 1 MMGH Consulting, Zurich, Switzerland; 2 Department of Infectious Disease Epidemiology, London School of Hygiene and Tropical Medicine, London, United Kingdom; 3 Department of Obstetrics and Gynaecology, Monash University, Melbourne, Australia; 4 Department of Immunization, Vaccines and Biologicals, World Health Organization, Geneva, Switzerland

**Keywords:** Group B *Streptococcus*, vaccination, maternal, antenatal care

## Abstract

**Background:**

To inform the World Health Organization’s full value of vaccine assessment for group B *Streptococcus* (GBS) vaccines, a rapid literature appraisal was conducted to inform the operationalization of maternal GBS vaccination. We found limited published information on stakeholder perceptions of the public health importance of GBS disease and vaccination, and we therefore undertook a multicountry survey.

**Methods:**

An online survey was conducted in late 2019 to collect information on stakeholders’ awareness of GBS disease and the priority accorded to vaccination. The survey was distributed by email to 395 representatives of national pediatric, gynecology, and obstetrics associations, national immunization technical advisory groups (NITAGs), national regulatory agencies, academia, and United Nations organizations.

**Results:**

Among 101 survey respondents from 66 countries, 36% were pediatricians, 25% obstetricians/gynecologists, 21% immunization specialists, and 18% other public health specialists. More than half (58%) of respondents reported being familiar with GBS disease as a public health problem; familiarity decreased by country income level. Knowledge of GBS disease was greatest in the Americas (68%) and Europe (66%) and lowest in Asia (13%–38%). Perception of GBS disease as a public health problem was highest among pediatricians (71%) and lowest among public health policy makers and NITAG members (30%) across country groupings. Approximately half of respondents (49%) considered the introduction of a GBS vaccine as a priority.

**Conclusions:**

The information obtained will inform the appropriate packaging and presentation of information to address stakeholder perceptions and promote evidence-based decision making on GBS vaccination.

**KEY FINDINGS**

1. What was known and what is new? Group B *Streptococcus* (GBS) is an important pathogen contributing to serious disease and death in women and their infants. There is limited information on stakeholder perceptions on the public health importance of GBS disease and the prioritization of its prevention through vaccination.
2. What did we do and what did we find? We conducted a global online survey of key stakeholders and influencers of vaccine policy making. A total of 101 individuals from 66 countries responded, and more than half had heard of GBS. Awareness of GBS disease was lowest in low-income countries and in Asia. Almost half of respondents perceived a GBS vaccine to be a priority, while gaps were reported in specific evidence to inform the next steps for national policy making and program development.
3. What to do now in programs? Ensure that stakeholder perceptions are considered in summarizing and presenting data for policy making and more investment in communicating existing evidence.
4. What next for research? Our survey focused on health professionals and policy makers, and similar surveys are needed to consider the views of potential vaccine recipients, notably pregnant women. In addition, important gaps in the data need to be addressed, regarding national estimates for GBS burden and their dissemination once a GBS vaccine is available.

Maternal immunization can play an important role in improving maternal, fetal, and neonatal health and is an important tool in the public health armamentarium. As a relatively low-cost intervention it can help low- and middle-income countries (LMICs) achieve the Sustainable Development Goals 3.1 and 3.2 [1]. New comprehensive estimates of the burden of group B *Streptococcus* (GBS) disease show that this pathogen causes serious consequences for women and their infants by contributing to stillbirths as well as infant deaths [*Placeholder: GBSSupplementPaper on burden of disease*]. New data also report the extensive impact of GBS disease on lifelong neurodevelopmental potential [*Placeholder: GBSSupplementPaper on NDI*]. Intrapartum antibiotic prophylaxis (IAP) is presently the only preventive strategy available. A World Health Organization (WHO) recommendation on IAP exists [2], but IAP policies and strategies are currently implemented almost exclusively in upper-middle-income countries (UMICs) and high-income countries (HICs) [3]. Many LMICs may lack the resources to establish the infrastructure and technical capacity to implement IAP.

Because of the large burden of disease and the technical feasibility of developing an effective vaccine, GBS has been identified as a high priority by the WHO Product Development for Vaccines Advisory Committee [4]. The impact of a maternal GBS vaccination program on maternal and infant outcomes will depend on the vaccine efficacy, timing of administration during pregnancy, and uptake. The WHO preferred product characteristics assume that the vaccine would be provided as a single dose during the second or third trimester of pregnancy to all pregnant women, irrespective of GBS colonization status or history of vaccination or infection.

To inform WHO’s full value of vaccine assessment for GBS vaccines, information to address issues related to implementation of vaccination, including policy making, service delivery, acceptance and uptake of vaccination, monitoring and evaluation, was collected through a rapid appraisal of the literature. The results of this review are described in a WHO document [5] that was developed through a consultative process with a WHO technical working group. The findings of the WHO report are briefly outlined below.

## POLICY FORMULATION

Establishing national policy recommendations for GBS vaccination is an essential first step for including the vaccine in a national immunization program (NIP) and making it available for pregnant women [6]. Many LMICs have national policies for maternal vaccination in place, mainly with tetanus toxoid containing vaccine [7] but increasingly also with seasonal influenza vaccine [8]. With a growing number of vaccines being added to the national immunization schedules in LMICs, governments are likely to require stronger justification for adding further vaccines to ensure that they are making appropriate investment choices in the face of competing public health priorities.

Systematic reviews that summarize available evidence on GBS disease and prevention have been updated [*Placeholder: GBSSupplementPaper*]. The economic burden of GBS disease and the potential benefits of vaccination are described in this supplement. While there are published estimates of stillbirths and the burden of GBS disease in children and pregnant women [9], there are still knowledge gaps, given a lack of comparable national level estimates for all relevant GBS disease outcomes. Data on the health and economic burden of disease and on stakeholder perceptions about the public health importance of GBS will play an important role in decision making and in building the economic case for investing in GBS vaccination.

## SERVICE DELIVERY

GBS vaccines targeting pregnant women may be delivered through either the NIP or the antenatal care (ANC) program, and possibly a combination of both. Opportunities and challenges for collaboration or integration of ANC and immunization services have been explored in the Maternal Immunization and Antenatal Care Situation Analysis project [10, 11]. More than half (54%) of the 116 LMICs in Africa, Asia, and Latin America assessed in this project were providing tetanus toxoid containing vaccines to pregnant women along with ANC [11]. Given the need to vaccinate pregnant women against GBS within a specific time frame during pregnancy, ANC visits may be better suited than the NIP to deliver vaccination if contact occurs in the second or third trimester. However, certain functions, such as vaccine procurement and supply chain management, may still be done by the NIP. 

The nature and quality of the collaborations between NIP and ANC services will need to be further strengthened for the timely delivery of GBS vaccination, including a clear definition of roles and responsibilities. One-stop approaches enabling maternal vaccination to take place simultaneously with ANC visits may hold the key to addressing shortcomings with the expansion of maternal immunization programs [10].

## ACCEPTANCE AND UPTAKE OF VACCINATION

Several factors in vaccine acceptance and uptake appear to be common across immunization and ANC programs—for example, the influence of health worker recommendations on vaccine uptake [12, 13]. However, many of these factors are contextual and vary both between and within countries, driven by local sociocultural practices, beliefs, and access to—and the quality of—healthcare services. Several tools exist to examine the drivers of vaccine uptake and tailor programs accordingly, which could be leveraged for GBS vaccination [14]. In 3 studies, all from HICs, a high proportion of women indicated their willingness to accept a hypothetical GBS vaccine, especially when information on GBS disease was provided [15–17].

## MONITORING AND EVALUATION

Almost all countries report vaccination coverage data collected through their national health management information systems (HMIS) or coverage surveys, and often from both sources [18]. Many LMICs also collect ANC data in their HMIS, and one or both systems can be leveraged to collect GBS vaccination data. Few LMICs have systems for collecting data on GBS outcomes to monitor impact, including invasive disease in neonates and infants, maternal sepsis, and stillbirths. However, the scope of the currently existing sentinel site surveillance networks could be expanded to collect information on neonatal and maternal sepsis [19]. Many LMICs do not have complete civil registration and vital statistics systems that collect stillbirth data. However, efforts to strengthen these systems provide opportunities to capture stillbirth rates and document the impact of vaccination on stillbirths in at least a representative set of countries [20].

Safety monitoring of maternal vaccination is complex and requires the monitoring of adverse events in pregnant women as well as adverse pregnancy outcomes. Few LMICs have well-functioning systems for monitoring the safety of maternal vaccination. Here again, opportunities exist that can be leveraged to strengthen safety monitoring. The Global Alignment of Immunization Safety Assessment in Pregnancy (GAIA) network has established case definitions and guidelines for monitoring adverse events, and a road map and technical assistance are available to LMICs to establish or strengthen their safety monitoring systems in preparation for GBS vaccination [21, 22].

In addition to data and evidence on the burden and epidemiology of disease and the safety and efficacy of vaccines, stakeholder perceptions of the public health importance of the targeted disease and of public acceptance of vaccination may also influence decisions on prioritizing a vaccine for inclusion in the national immunization schedule. Since these data were not available in the literature, an online survey was conducted to collect information on the existing level of stakeholders’ awareness of GBS disease, the disease outcomes that would influence policy decisions, the management of GBS disease and its prioritization in different countries, and the potential uptake of vaccination.

## AIM AND OBJECTIVES

This article is the part of a series from this GBS supplement. To inform WHO’s full value of vaccine assessment for GBS vaccines, it aims to (1) summarize key considerations for the operationalization of maternal GBS vaccination in LMICs and for achieving their immunization goals and (2) fill information gaps on stakeholder perceptions of the public health importance of GBS disease and vaccination, using data from a multicountry survey.

## METHODS

An online survey was conducted in late 2019 using a standardized questionnaire covering the following areas: (1) awareness of GBS disease; (2) awareness and use of GBS screening and IAP; (3) knowledge of clinical manifestations and GBS disease outcomes; (4) perception of GBS disease as a public health problem; and (5) perceptions of the need for, and priority of, prevention strategies. The survey also explored existing country policies and approaches to screening and IAP, including counseling in ANC services, coverage, and barriers to implementation of IAP. Finally, respondents were asked to provide their thoughts about potential future GBS vaccines, their prioritization and anticipated level of acceptance, and potential barriers to vaccine implementation. The survey tool was newly developed and consisted of 23 multiple-choice, Likert scale–ranked, and free text questions in the English language (see Supplementary Materials for survey questionnaire and full breakdown of survey responses).

The stakeholder survey targeted representatives of national pediatric associations, gynecology and obstetrics associations, NITAGs, national regulatory agencies, academia, and United Nations agencies. These included pediatricians, obstetricians, immunization specialists and public health policy makers residing and working in countries representative of all World Bank income groups. Respondents were approached via email addresses obtained from full membership lists of the International Paediatric Association and the International Federation of Gynaecology and Obstetrics. In addition, academic institutions, immunization partner institutions, vaccine manufacturer associations, and UN health agencies in all WHO Regions were contacted using a database held by MMGH Consulting. 

The survey was distributed to 420 email addresses via the online survey tool Qualtrics (CoreXM online platform); 25 addresses were not reachable. Two reminders were sent to respondents. Descriptive analysis of the survey responses was done using MS Excel software, with stratification of quantitative responses by different domains (eg, professional association, world region, and country income group). A brief thematic analysis was performed on responses to the open-ended survey questions, using an inductive coding approach. By participating in the survey, respondents explicitly agreed to the processing of their responses in line with MMGH’s data privacy policy. The data sets used and analyzed during the current study are available from the corresponding author on reasonable request.

## RESULTS

Of 395 individuals from 66 countries who received the email, 101 (26%) responded to the survey. Table 1 provides an overview of the response rate according to stakeholder group, country income group and WHO region. Further analysis includes the 101 individuals who provided full or partial responses (respondents).

**Table 1.** Survey Response Rates by Stakeholder Group, Country Income Level, and WHO Region

Category	Surveys Sent, No.	Full and Partial Responses, No.	Response Rate, %
Stakeholder group^a^			
Pediatrics/ obstetrics-gynecology	259	54	20
Public health policy	52	19	37
Academic/research	26	7	27
Government institutions	20	8	40
Implementing partners (eg, NGOs/UN)	33	9	27
Industry/ manufacturers	5	4	80
Country income level			
LICs	38	10	26
LMICs	88	18	20
UMICs	99	22	22
HICs	169	51	30
WHO region			
Africa	67	20	30
Americas	67	19	28
Eastern Mediterranean	37	7	19
Europe	146	39	26
South-East Asia	34	8	24
Western Pacific	42	8	19

Abbreviations: HICs, high-income countries; LICs, low-income countries; LMICs, low- and middle-income countries; NGOs, nongovernmental organisations; UMICs, upper-middle-income countries; UN, United Nations; WHO, World Health Organization.

^a^Public health policy includes members of WHO’s Strategic Advisory Group of Experts on Immunization (SAGE) and members of national immunization technical advisory groups and regional immunization technical advisory groups. Government institutions include national immunization program managers. Those in the pediatrics/obstetrics-gynecology group were all members of their respective national associations, and it is likely that most were physicians.

Among the respondents 36% were pediatricians, 25% obstetricians/gynecologists, 21% immunization specialists, and 18% other public health specialists. Approximately half of the respondents were from HICs, 40% from middle-income countries, and 10% from low-income countries (LICs). Thirty-nine percent of respondents worked in countries in the WHO European Region, 20% in the African Region, 19% in the Region of the Americas, and slightly less than 10% each in the Eastern Mediterranean, South-East Asia, and Western Pacific regions, respectively.

More than half (58%) of respondents considered themselves to be very familiar with GBS disease as a public health problem. This familiarity, however, varied by country income level, being highest in HICs and lowest in LICs (70%, 52%, 47%, and 30% in HICs, UMICs, LMICs, and LICs). Knowledge of GBS disease also varied by WHO region, being greatest in the Americas (68%), Europe (66%), and Africa (55%) and lowest in Asia (38% in the Western Pacific and 13% in South-East Asia).

**Figure 1.**
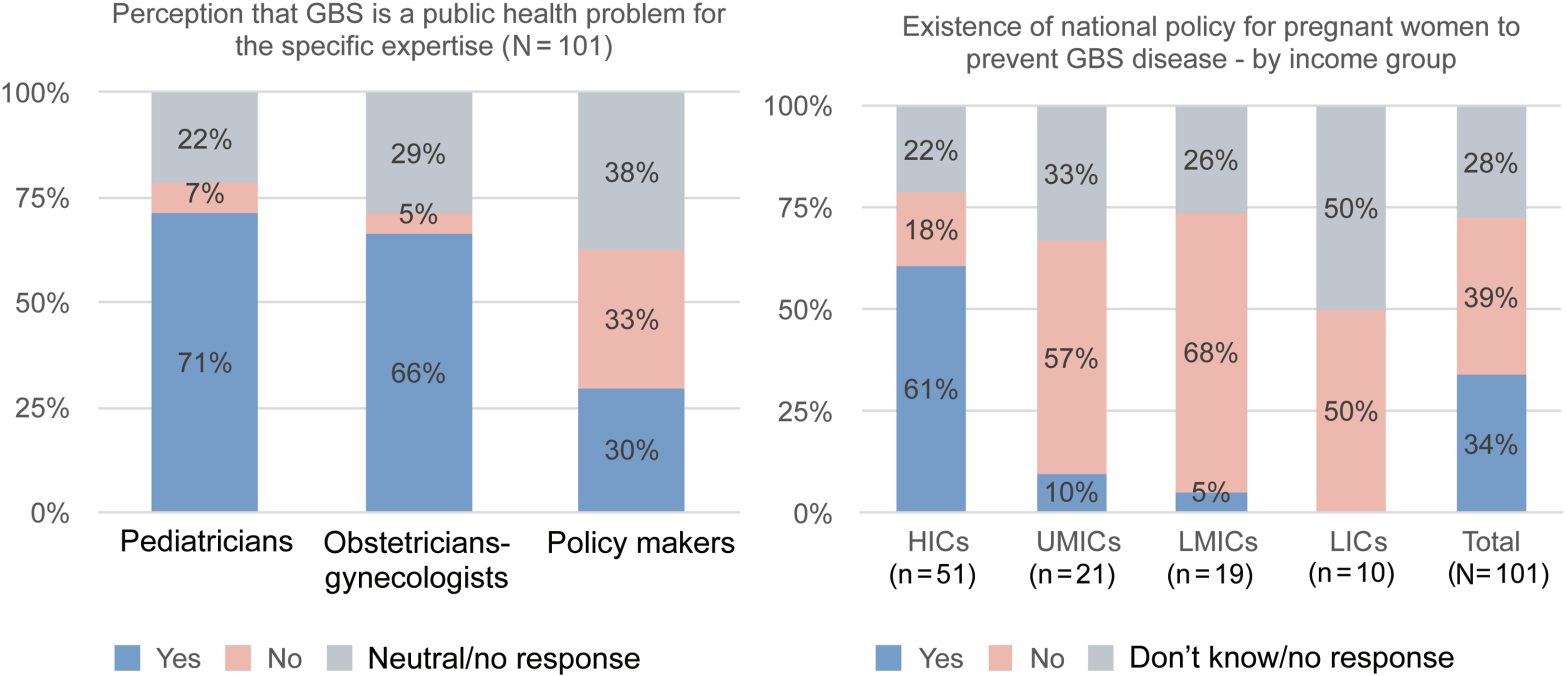
Perception that group B *Streptococcus* (GBS) disease is a public health problem and existence of national policy to prevent neonatal GBS disease. Abbreviations: HICs, high-income countries; LICs, low-income countries; LMICs, low- and middle-income countries; UMICs, upper-middle-income countries.

Stakeholders were asked their opinions about whether pediatricians, obstetricians, and policy makers considered GBS to be a public health priority in their respective countries. The survey responses (Figure 1) indicated that the perception of GBS disease as a public health problem was highest among pediatricians (71%) across all country groupings (78%, 52%, 68%, and 80% in HICs, UMICs, LMICs, and LICs) and WHO regions (38%–75%). 

The perception among obstetricians that GBS was a public health problem was estimated to be 66%; this perception was high across all country groupings (78%, 33%, 74%, and 60% in HICs, UMICs, LMICs, and LICs). In contrast, the perception among policy makers and NITAG members that GBS was a public health problem was only 30% (37%, 19%, 26%, and 20% in HICs, UMICs, LMICs, and LICs). In HICs, only 37% of respondents reported that policy makers in their countries considered GBS aa public health problem. Thirty-four percent of respondents indicated that their countries had a national policy or guideline to prevent GBS disease in pregnant women. However, this percentage again differed substantially by income group (61%, 10%, 5%, and 0% in HICs, UMICs, LMICs, and LICs).

Further qualitative analysis of open responses indicated that respondents considered pediatricians—and specifically neonatologists—to be generally aware and knowledgeable of the disease. However, they also stated that GBS detection was not routinely done in many countries and that culture results even in suspected cases were often negative, partly owing to the indiscriminate use of antibiotics before admission or because of absent or inadequate laboratory infrastructure. 

Respondents from several regions were also concerned about the absence of systematic population-based studies to document the burden of GBS disease. Several respondents (n = 34) stated that this lack of data limited their ability to convince public health decision makers of the extent of the problem, including its economic impact. Several respondents from Asia considered the prevalence of GBS neonatal sepsis to be lower in Asia than in Western countries (eg, in Europe and North America). Consequently, in the absence of adequate local data, they did not believe that routine screening of pregnant women would be beneficial in their countries.

**Figure 2.**
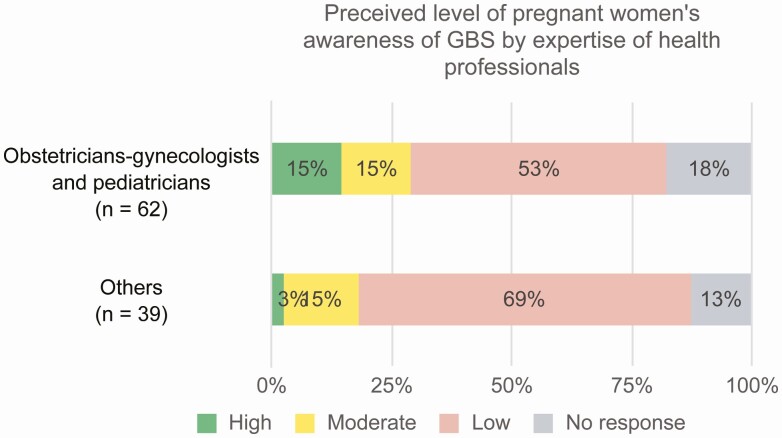
Perceived level of awareness of pregnant women of group B *Streptococcus* (GBS) disease, as reported by health professionals and policy makers. “Others” include public health policy and immunization experts.

When asked to estimate the degree of GBS awareness and knowledge among women, respondents stated that about one-quarter of pregnant women would have some degree of such awareness, and this did not vary substantially between clinicians (pediatricians and obstetricians) and other respondents (Figure 2). A stark difference is apparent here between country groupings; while in HICs half of the pregnant women were considered somewhat familiar with the disease, only 18% were deemed aware of GBS disease in UMICs, and none in the lower-income countries. Respondents from the WHO South-East Asia, Western Pacific, and African regions thought that pregnant women in their respective countries were likely to be completely unaware of GBS disease. 

A similar picture emerged when respondents were asked about the proportion of pregnant women who would be counseled about GBS disease during ANC visits. Based on the survey results, only 23% of pregnant women globally were expected to receive such counseling, including almost half of pregnant women in HICs (41%) but only 10% in UMICs and none in the LMICs and LICs. Responses indicated that about 30%–40% of women received counseling in Europe, the Americas, and the Western Pacific Region, but respondents were not aware of counseling being performed in the other 3 regions. While the responses are as anticipated from LICs and LMICs, where preventive interventions during pregnancy are not generally offered, the relatively low rates of counseling in higher-income settings are notable.

**Figure 3.**
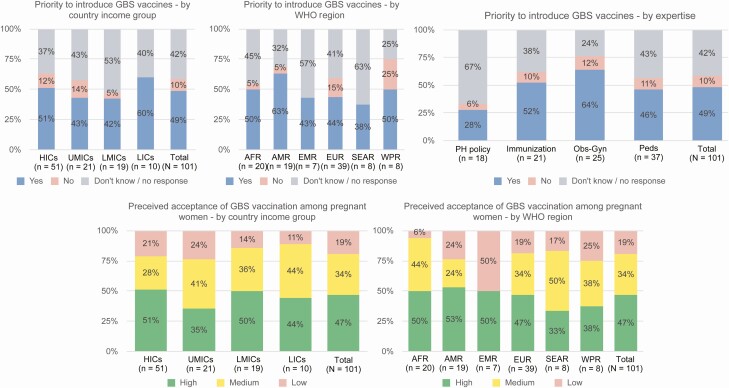
Respondents’ view of group B *Streptococcus* (GBS) vaccine prioritization and of perceived acceptance of GBS vaccination among pregnant women, as reported by health professionals and policy makers. Abbreviations: AFR, African Region; AMR, Region of the Americas; EMR, Eastern Mediterranean Region; EUR, European Region; HICs, high-income countries; LICs, low-income countries; LMICs, low- and middle-income countries; Obs-Gyn, obstetricians/gynecologists; Peds, pediatricians; PH, public health; SEAR, South-East Asia; UMICs, upper-middle-income countries; WPR, Western Pacific Region.

Approximately half of survey respondents (49%) considered the introduction of a GBS vaccine a priority, with >60% of obstetricians/gynecologists, 46% of pediatricians, and >50% of immunization specialists indicating that they considered it a priority. About half of respondents were confident that pregnant women would accept a GBS vaccine. This confidence, however, was supported by fewer respondents in Asia and in UMICs (Figure 3).

The public health arguments (based on national public health priorities) that respondents believed would support a policy decision for the introduction of a GBS vaccine included (in order of priority): (1) reduction of neonatal mortality rates by preventing sepsis; (2) reduction in stillbirths; (3) reduction in maternal sepsis; (4) the perceived cost-effectiveness of vaccination versus treatment of invasive disease; (5) reduction in long-term impairment; and (6) the perceived high acceptability of vaccination among pregnant women and ease of including a GBS vaccine in existing immunization services. 

**Figure 4.**
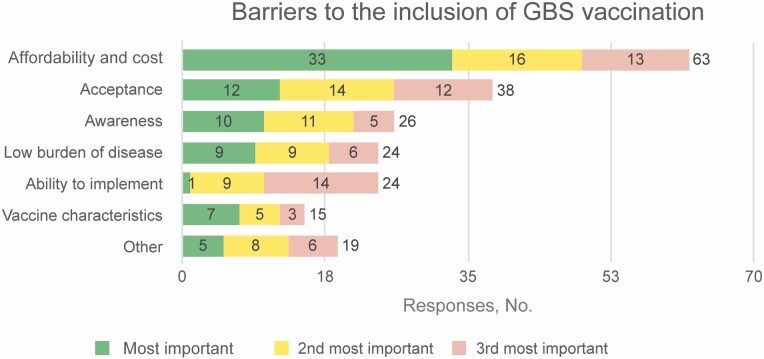
Respondents’ view of barriers to inclusion of group B *Streptococcus* (GBS) vaccination in national immunization programs.

The main barriers to introduction of a GBS vaccine were deemed to be vaccine costs and affordability, including cost-effectiveness considerations, lack of acceptance and awareness of the disease or vaccine, lack of reliable data on or low prevalence of GBS disease, the inability to implement vaccination (eg, low capacity, no platform for routine immunization of pregnant women, and low ANC coverage), vaccine characteristics (eg, efficacy, effectiveness, and safety) and other issues (eg, competing priorities and use of alternative prevention methods, such as IAP) (Figure 4).

## Discussion

Policy recommendations are an important first step for the introduction of any new vaccine into the national immunization schedule. Policy making should ideally be based on evidence of the burden of disease, the safety and efficacy of vaccines, and the cost-effectiveness and budget impact of vaccination programs. However, previous experience with other vaccines, most notably the *Haemophilus influenzae* type b vaccine, has demonstrated that merely providing estimates of cases and deaths derived from mathematical models may not be sufficient to convince public health policy makers to prioritize a vaccine for inclusion in the national vaccination schedule [23]. National policy makers are likely to seek local data to justify the inclusion of vaccines, especially in light of the perceptions of low public priority of a disease such as GBS among key opinion leaders and as national budgets for immunization programs increase and LMICs transition out of support from Gavi, the Vaccine Alliance. Stakeholder perceptions of the public health importance of a disease and its prevention through vaccination can thus be important drivers of country decision making.

This stakeholder survey provides some useful preliminary insights into such perceptions. While the survey showed a reasonably high level of awareness of GBS disease and its prevention among pediatricians and obstetricians in all WHO regions and in countries with different income levels, awareness among public health specialists was low, especially in LMICs and LICs. Such awareness in the public health community must be raised through information and advocacy efforts.

The limited laboratory capacity in LMICs may be a contributing factor to the low recognition of GBS as a public health problem. Although neonatal sepsis is considered an important cause of neonatal deaths, in the absence of adequate laboratory capacity, treatment guidelines often promote the use of syndromic diagnosis, without microbiological confirmation [24]. As a consequence, the low rate of GBS detection seems to have contributed to a low proportion of LMICs and LICs with national policies or guidelines for counseling and provision of IAP. Similarly, unless greater efforts are made to improve the detection of GBS disease in at least a representative sample of sites in LMICs, the lack of empirical data is likely to hinder decisions on vaccine introduction.

Although a high proportion of stakeholders in HICs were familiar with GBS disease, opinions were voiced that public health policy makers in these countries still did not consider GBS a public health problem. This could be influenced by the fact that IAP is being implemented in most of these countries, reducing the recognizable burden of GBS disease.

The perceived low rates of awareness of GBS disease in pregnant women is concerning, especially in lower-income countries, and will need to be further elucidated in country-based studies. Adapting conceptual frameworks used in HICs for use in LMICs could help generate local data on factors that raise awareness of the risk of GBS disease and enable countries to provide relevant information to pregnant women, sufficiently in advance of vaccine introduction.

The main barriers to vaccine introduction were considered cost and affordability. To address this barrier, evidence and information on GBS disease should be contextualized to national public health priorities. Reduction in neonatal deaths and stillbirths were the 2 public health arguments that were most often mentioned to promote the use of GBS vaccination. Hence, it is important to appropriately package information and advocacy materials targeted at public health policy makers. However, once again, it will not suffice to make such arguments based purely on modeled data without any supportive local evidence. Even in the absence of data from active community-based surveillance, a compilation of local data from hospital-based studies may be used to complement the modeled estimates of cases and deaths.

Although the survey provided some first-hand data on the awareness and perceptions on the GBS burden of disease and the potential acceptance of vaccination, it had several limitations. The response rate of 26%, albeit limited, is consistent with rates of similar global online surveys. However, selection bias could have been introduced, and findings may not be representative of all global stakeholder views. For logistical reasons, the online survey was limited to professional groups involved in policy setting and implementation. The perceptions and views on GBS disease and vaccination of the health worker community and of pregnant women as potential vaccine recipients were not captured but could provide useful additional information relevant for formulating vaccination policy and delivery strategies in LMICs. Additional in-depth interviews with health professionals (eg, midwives and nurses) and women of childbearing age will therefore be helpful.

In conclusion, while there are challenges with the implementation of GBS vaccination, several existing opportunities can be leveraged to enable the successful introduction of the vaccine in LMICs. However, for a vaccine to be prioritized for inclusion in NIPs, it is important that stakeholder perceptions on the public health importance of GBS disease and the potential for vaccination are well addressed. The information obtained from the survey will enable the appropriate packaging and presentation of information to address stakeholder perceptions and promote well-informed decision making (see also Box 1).

## Supplementary Data

Supplementary materials are available at *Clinical Infectious Diseases* online. Consisting of data provided by the authors to benefit the reader, the posted materials are not copyedited and are the sole responsibility of the authors, so questions or comments should be addressed to the corresponding author.

ciab794_suppl_Supplementary_Materials

## Notes

***Acknowledgments.*** We express appreciation to the GBS scientific advisory group and technical working group members: Martina Lukong Baye, Lumbwe Chola, Michelle Giles, Paul T. Heath, Margaret Ip, Shabir A. Madhi, Nicholas Kassebaum, Asma Khalil, Kirsty Le Doare, Sonja Merten, Flor M. Munoz, Maarten Postma, Samir Saha, Stephanie Schrag, and Baoping Yang.

***Author contributions.*** C. M. and T. C. conceptualized the group B *Streptococcus* study. C. M., together with T. C., S. M., and P. L., developed the detailed research questions and the overall analysis plan for this article. These were refined with inputs from E. M. and M. G. Analysis was undertaken by C. M., T. C., and M. K. The manuscript was drafted by C. M. and T. C. All authors reviewed and helped revise the manuscript and agreed on the final version.

***Supplement sponsorship.*** This supplement is sponsored by Bill & Melinda Gates Foundation.

***Financial support.*** This work is supported by the Bill & Melinda Gates Foundation (grants INV-1175247 to the WHO Immunization, Vaccines and Biologicals department, INV-009018 to the London School of Hygiene &Tropical Medicine, OPP1180644) to WHO and the London School of Hygiene & Tropical Medicine, and INV-007608 to WHO); and WHO (support to MMGH Consulting through purchase orders 202309505 and 202482293; support to C. M. and T. C. through MMGH Consulting).

***Potential conflicts of interest.*** All authors: No reported conflicts. All authors have submitted the ICMJE Form for Disclosure of Potential Conflicts of Interest. Conflicts that the editors consider relevant to the content of the manuscript have been disclosed.
