# Supplementary material for: Stakeholder Perceptions About Group B Streptococcus Disease and Potential for Maternal Vaccination in Low- and Middle-Income Countries
Source: Clin Infect Dis. 2021 Nov 3;74(Suppl 1):S80–7. doi: 10.1093/cid/ciab794 (PMC8776310; doi:10.1093/cid/ciab794)
Supplement: ciab794_suppl_Supplementary_Materials [file ciab794_suppl_supplementary_materials.docx]

**SUPPLEMENTARY MATERIAL**

**Supplement Title:** Every Country, Every Woman, Every Child; Group B Streptococcal Disease Worldwide

**Paper Title:** Stakeholder perceptions about Group B Streptococcus disease and its prevention through vaccination in low and middle income countries

**Authors:** Carsten Mantel^1*^, Thomas Cherian^1^, Melissa Ko^1^, Stefano Malvoti^1^, Elizabeth Mason^2^, Michelle Giles^3^, Philipp Lambach^4^

**Affiliations:**

1. MM Global Health Consulting, Zurich, Switzerland
2. Department of Infectious Disease Epidemiology, London School of Hygiene and Tropical Medicine, London, UK
3. Department of Obstetrics and Gynaecology, Monash University, Melbourne, Australia
4. Department of Immunization, Vaccines and Biologicals, World Health Organization, Geneva, Switzerland.

**Corresponding Author** Carsten Mantel, Managing Director, MMGH Consulting GmbH, Kürbergstr. 1, 8049 Zürich, Switzerland, [mantelc@mmglobalhealth.org](mailto:mantelc@mmglobalhealth.org)

Table of Contents

[Annex: Stakeholder survey on group B streptococcal (GBS) disease and vaccine prioritization 3](#_Toc81559767)

[Supplementary Materials 10](#_Toc81559768)

# Annex: Stakeholder survey on group B streptococcal (GBS) disease and vaccine prioritization

Q1 Dear Colleague,

This survey is organized as part of an investigative work organized for the World Health Organization, Geneva and under the auspices of a Scientific Advisory Group which provides inputs to the development of a Group B Streptococcal (GBS) vaccine value proposition.

The goal of this questionnaire is to investigate the existing level of awareness of GBS disease in your country and how GBS vaccination would likely be prioritized in the future. As an important stakeholder whose thoughts, ideas and expert advice will be of utmost importance to the success of this process, we would like ask you to complete this survey by responding to its questions related to GBS disease, national policies and guidelines, existing GBS prevention strategies, screening and prophylaxis approaches and the potential acceptance of GBS vaccination among pregnant women in your country.

Privacy Policy

In addition to your views, we will be collecting demographic information on your organizational affiliation and employment location. That information will allow us to perform stratified analyses that will provide valuable insights. Your data are stored in data servers with MMGH’s provider of cloud services (Microsoft) and with our survey platform (Qualtrics). Those services are compliant with the European Union’s General Data Protection Regulations. Upon completion of the project, all demographic information will be deleted. Furthermore, all information collected will be anonymized and aggregated prior to being included into analyses and / or reports. The same data treatment (full anonymisation and disclosure of aggregated data only) will apply in the event of publication of the results of this project.

Privacy References

US CDC privacy policy: https://www.cdc.gov/other/privacy.html

MMGH’s privacy policy: https://mmglobalhealth.org/privacy-policy/

Microsoft’s privacy policy: https://privacy.microsoft.com/en-us/privacystatement

Qualtrics’s privacy policy: https://www.qualtrics.com/privacy-statement/

By clicking the arrow button, you agree that MMGH will process your responses in line with its privacy policy stated above. If you have any questions you can contact our Data Protection Officer: Melissa Ko (kom@mmglobalhealth.org)

Your answers will be automatically saved in the cloud once provided. This may take some time depending on the speed of your internet connection and we kindly ask for your patience. Please note that the survey can be interrupted at any time and will remain active for one week from the first login. At the subsequent login you will be taken back to where you left off. 
 
 You can now start the survey by moving to the next page. Thanks in advance for your contribution.

Q2 What is your area of expertise/work?

- Public health and policy (1)
- Paediatrics (2)
- Obstetrics (3)
- Antenatal care (4)
- Immunization (5)
- Other (please specify) (6) ________________________________________________

Q3 What is your current level of awareness of GBS as a public health problem?

- Extremely familiar (1)
- Very familiar (2)
- Moderately familiar (3)
- Slightly familiar (4)
- Not familiar at all (5)

Q4 Do you consider GBS disease a public health problem in your country?

- Yes (1)
- No (2)

Q5 In your opinion, do paediatricians, obstetricians, public health policy-makers in your country perceive GBS disease as a public health problem?

|  | Strongly agree (11) | Somewhat agree (12) | Neither agree nor disagree (13) | Somewhat disagree (14) | Strongly disagree (15) |
| --- | --- | --- | --- | --- | --- |
| Pediatricians (1) |  |  |  |  |  |
| Obstetricians (2) |  |  |  |  |  |
| Public health policy-makers (6) |  |  |  |  |  |

Q6 Please summarize the possible reasons for your response

________________________________________________________________

Q7 Are you aware of the GBS disease manifestations and/or outcome in pregnant women, neonates and/or infants?

- Yes (1)
- No (2)

Q8 If yes, please list up to five GBS disease manifestations or outcomes that you are aware of in pregnant women, neonates and/or infants (once listed, please attribute them a ranking between 1 and 5).

______ Click to write Item 1 (1)

______ Click to write Item 2 (2)

______ Click to write Item 3 (3)

______ Click to write Item 4 (4)

______ Click to write Item 5 (5)

Q9 Are you aware of any GBS infection and disease prevention strategies in pregnant women?

- Yes (1)
- No (2)

Q10 If yes, please describe:

________________________________________________________________

Q11 Are you aware of any GBS infection and disease prevention strategies in neonates?

- Yes (1)
- No (2)

Q12 If yes, please describe

________________________________________________________________

Q13 Does your country have a national policy or guideline for pregnant women to prevent neonatal GBS disease?

- Yes (1)
- No (2)
- Do not know (3)

Q14 Is screening for GBS in pregnant women and subsequent antibiotic prophylaxis — if GBS screening is positive — performed in your country?

- Yes (1)
- No (2)
- Do not know (3)

Q15 If GBS screening is performed, what would is your estimate of the coverage of GBS screening? (please drag the slider to indicate your coverage estimate)

|  | 0 | 10 | 20 | 30 | 40 | 50 | 60 | 70 | 80 | 90 | 100 |
| --- | --- | --- | --- | --- | --- | --- | --- | --- | --- | --- | --- |

| Coverage () | 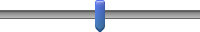 |
| --- | --- |

Q16 Are there any actual or potential barriers to screening pregnant women for GBS in your country?

- Yes (1)
- No (2)

Q17 If yes, please list the three most important barriers (once listed, please attribute them a ranking between 1 and 3).

______ Click to write Item 1 (1)

______ Click to write Item 2 (2)

______ Click to write Item 3 (3)

Q18 In your opinion, what is the level of awareness of pregnant women about GBS disease in your country?

- Extremely familiar (1)
- Very familiar (2)
- Moderately familiar (3)
- Slightly familiar (4)
- Not familiar at all (5)

Q19 Are pregnant women routinely counselled about GBS disease during antenatal visits?

- Yes (1)
- No (2)
- Do not know (3)

Q20 A GBS vaccine is currently in development to be given to pregnant women. Would a GBS vaccine be a priority to introduce in your country if available at an acceptable cost?

- Yes (1)
- No (2)
- Do not know (3)

Q21 In your opinion what would be the level of acceptance of GBS vaccination among pregnant women in your country?

- Extremely fast (1)
- Somewhat fast (2)
- Average (3)
- Somewhat slow (4)
- Extremely slow (5)

Q22 Please rate the following items in terms of importance for public health policy-makers in your country in order to prioritize GBS vaccination for inclusion in the national immunization schedule (rank in order of importance by dragging the various boxes in order – a first move is required to make the ranking numbers appear)

______ Contribution to reduction of neonatal sepsis (1)

______ Contribution to reduction of neonatal mortality (2)

______ Contribution to reduction of still-births (3)

______ Contribution to reduction of maternal sepsis (4)

______ Contribution to reduction of long-term impairment (5)

______ Cost-effectiveness of vaccination versus treatment (6)

______ Ease of inclusion in national immunization programmes (7)

______ Vaccine acceptability among pregnant women (8)

Q23 Please list the 3 most important barriers to inclusion of GBS vaccination in the national immunization programme in your country (once listed, please attribute them a ranking between 1 and 3).

______ Click to write Item 1 (1)

______ Click to write Item 2 (2)

______ Click to write Item 3 (3)

Q24 What could be some key communication messages to convey the importance of GBS disease and its prevention in the general population?

______ Click to write Item 1 (1)

______ Click to write Item 2 (2)

______ Click to write Item 3 (3)

Thank you so much for your important input!

# Supplementary Materials

**
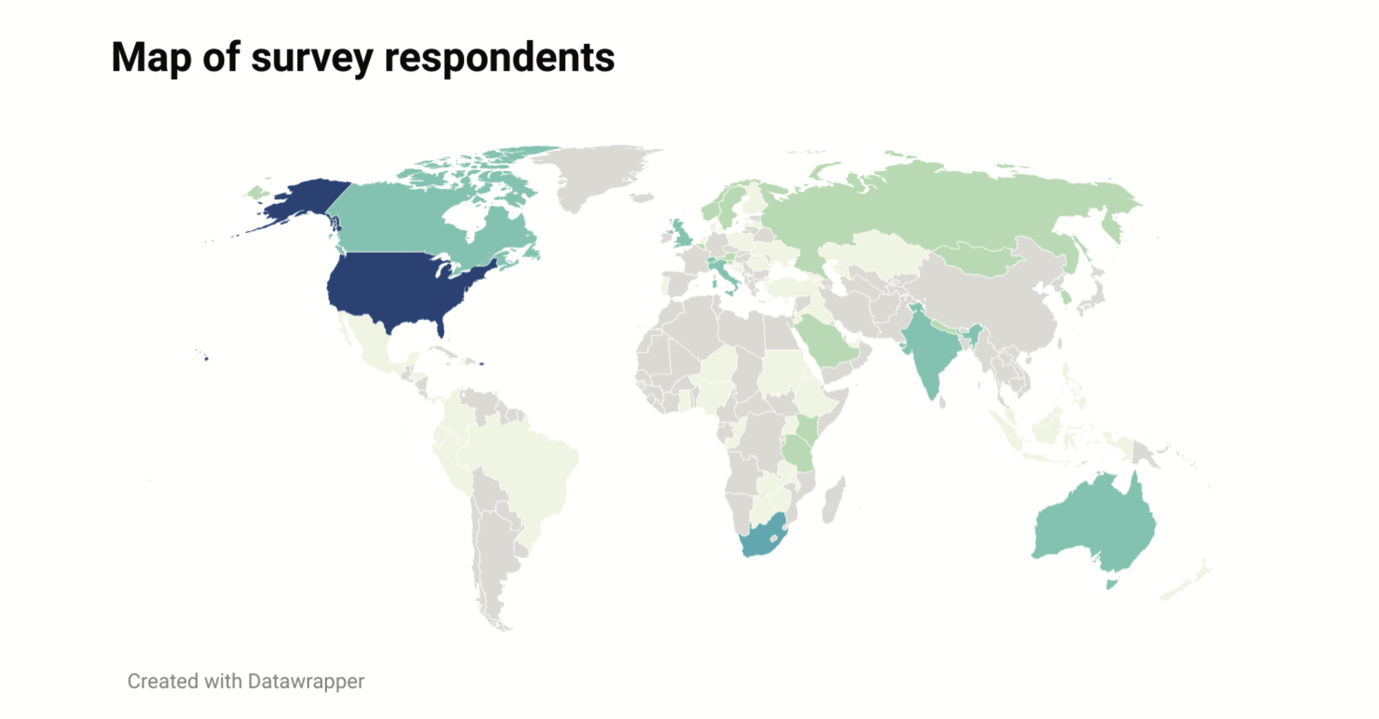
**

**
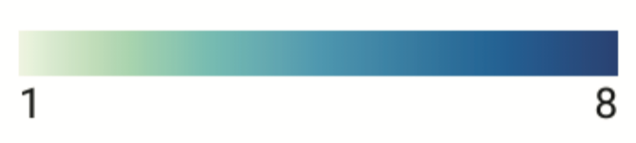
**

**Figure 5. Legend**

| **What is your current level of awareness of GBS as a public health problem?** | | |
| --- | --- | --- |
|  | # | % |
| Extremely familiar | 21 | 21% |
| Very familiar | 37 | 37% |
| Moderately familiar | 28 | 28% |
| Slightly familiar | 11 | 11% |
| Not familiar at all | 3 | 3% |
| No response | 1 | 1% |
| **Do you consider GBS disease a public health problem in your country?** | | |
|  | # | % |
| Yes | 69 | 68% |
| No | 21 | 21% |
| No response | 11 | 11% |
| **In your opinion, do the following individuals in your country perceive GBS disease as a public health problem?** | | |
|  | **Pediatricians** | |
|  | **#** | **%** |
| Agree | 72 | 71% |
| Neutral | 11 | 11% |
| Disagree | 7 | 7% |
| No response | 11 | 11% |
|  | **Obstetricians** | |
|  | **#** | **%** |
| Agree | 67 | 66% |
| Neutral | 16 | 16% |
| Disagree | 5 | 5% |
| No response | 13 | 13% |
|  | **Public-health policy makers** | |
|  | **#** | **%** |
| Agree | 30 | 30% |
| Neutral | 26 | 26% |
| Disagree | 33 | 33% |
| No response | 12 | 12% |
| **Are you aware of the GBS disease manifestations and/or outcome in pregnant women, neonates and/or infants?** | | |
|  | **#** | **%** |
| Yes | 90 | 89% |
| No | 4 | 4% |
| No response | 7 | 7% |
| **Are you aware of any GBS infection and disease prevention strategies in pregnant women?** | | |
|  | **#** | **%** |
| Yes | 74 | 73% |
| No | 14 | 14% |
| No response | 13 | 13% |
| **Are you aware of any GBS infection and disease prevention strategies in neonates?** | | |
|  | **#** | **%** |
| Yes | 34 | 34% |
| No | 39 | 39% |
| Don't know / no response | 28 | 28% |
| **Does your country have a national policy or guideline for pregnant women to prevent neonatal GBS disease?** | | |
|  | **#** | **%** |
| Yes | 34 | 34% |
| No | 39 | 39% |
| Don't know / no response | 28 | 28% |
| **Is screening for GBS in pregnant women and subsequent antibiotic prophylaxis, if GBS screening is positive, performed in your country?** | | |
|  | **#** | **%** |
| Yes | 44 | 44% |
| No | 35 | 35% |
| Don't know / no response | 22 | 22% |
| **Are there any actual or potential barriers to screening pregnant women for GBS in your country?** | | |
|  | **#** | **%** |
| Yes | 50 | 50% |
| No | 37 | 37% |
| Don't know / no response | 14 | 14% |
| **In your opinion, what is the level of awareness of pregnant women about GBS disease in your country?** | | |
|  | **#** | **%** |
| High | 10 | 10% |
| Moderate | 15 | 15% |
| Low | 60 | 59% |
| No response | 16 | 16% |
| **Are pregnant women routinely counselled about GBS disease during antenatal visits?** | | |
|  | **#** | **%** |
| Yes | 23 | 23% |
| No | 38 | 38% |
| Don't know / no response | 40 | 40% |
| **A GBS vaccine is currently in development to be given to pregnant women. Would a GBS vaccine be a priority to introduce in your country if available at an acceptable cost?** | | |
|  | **#** | **%** |
| Yes | 49 | 49% |
| No | 10 | 10% |
| Don't know / no response | 42 | 42% |
| **In your opinion what would be the level of acceptance of GBS vaccination among pregnant women in your country?** | | |
|  | **#** | **%** |
| High | 39 | 47% |
| Medium | 28 | 34% |
| Low | 16 | 19% |

**Table 2. Detailed responses for all survey questions**
